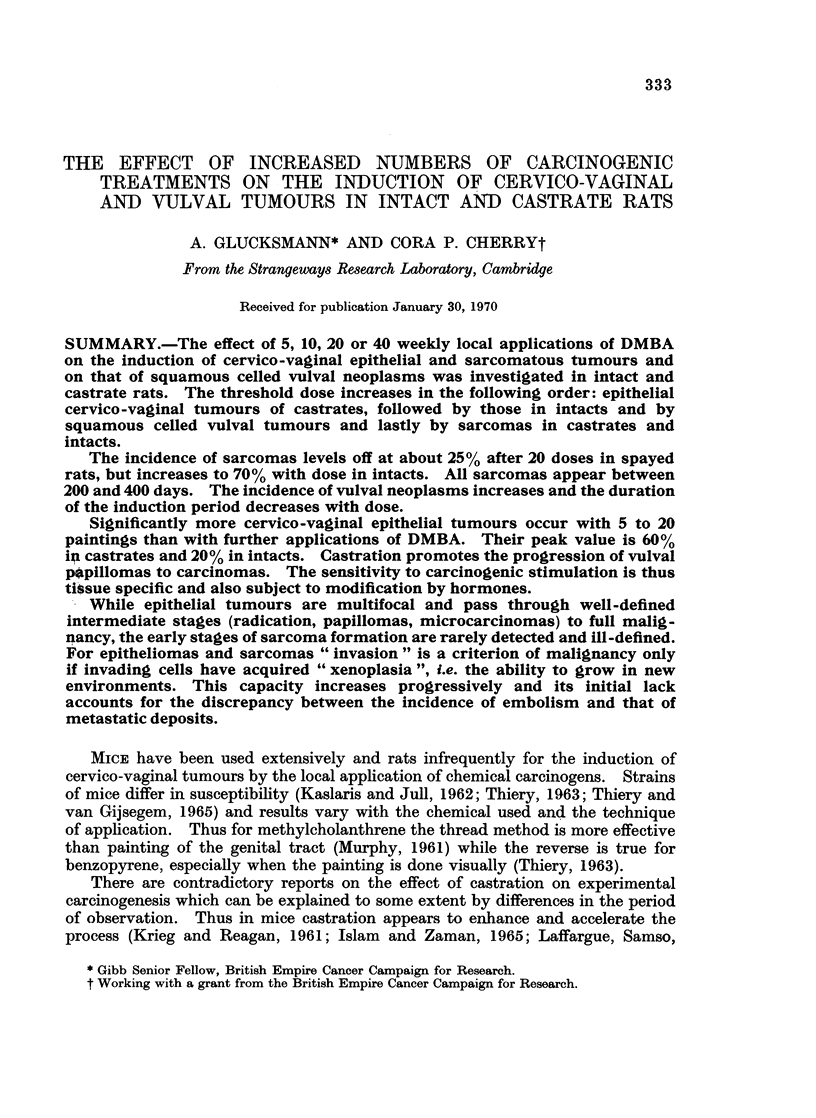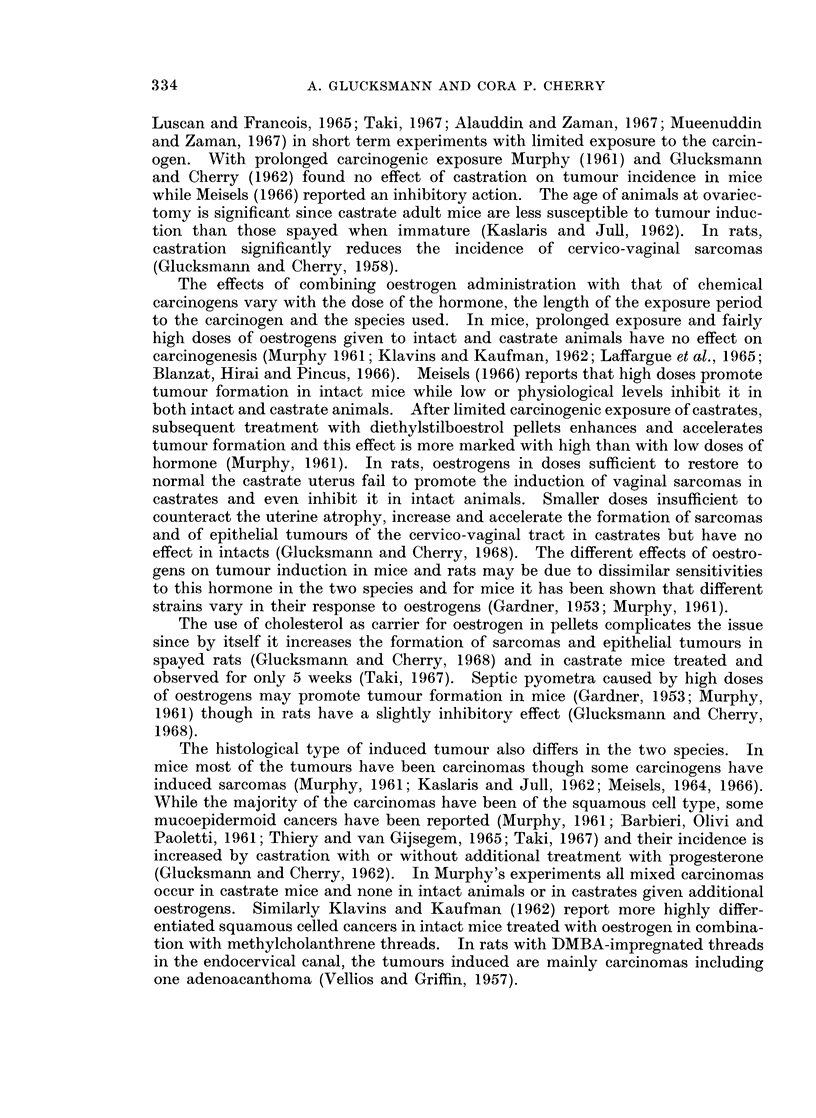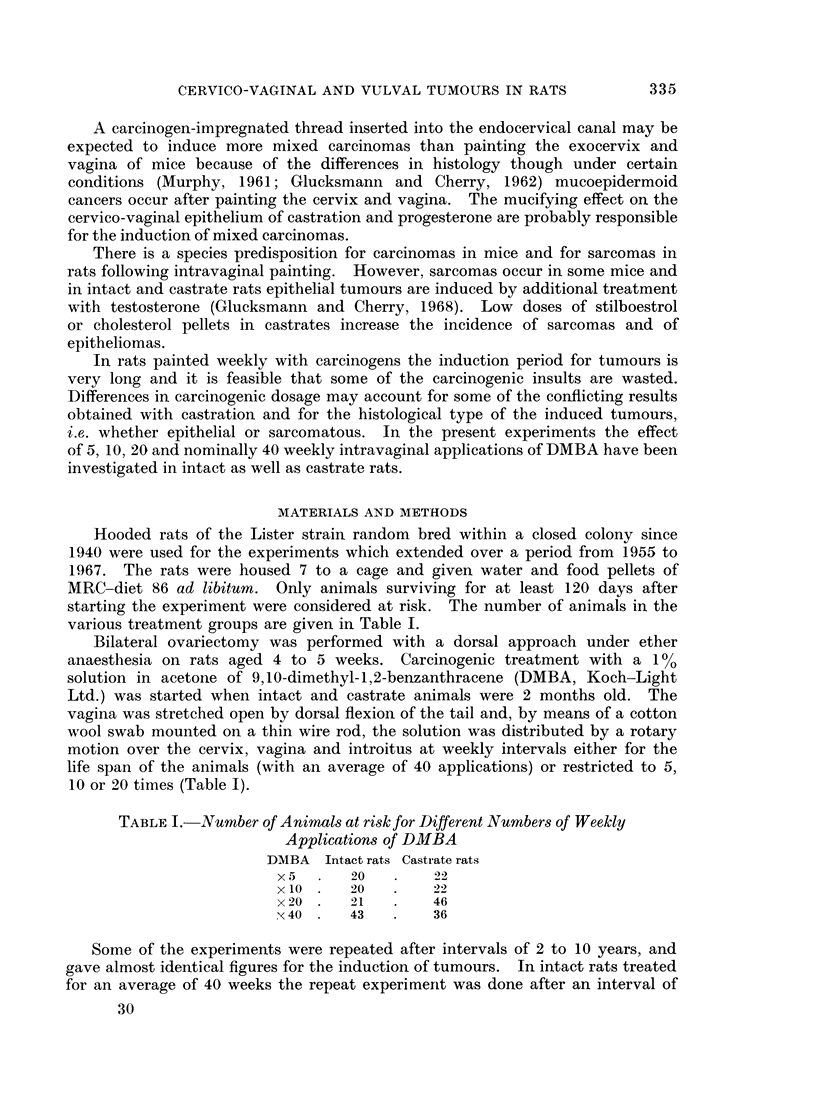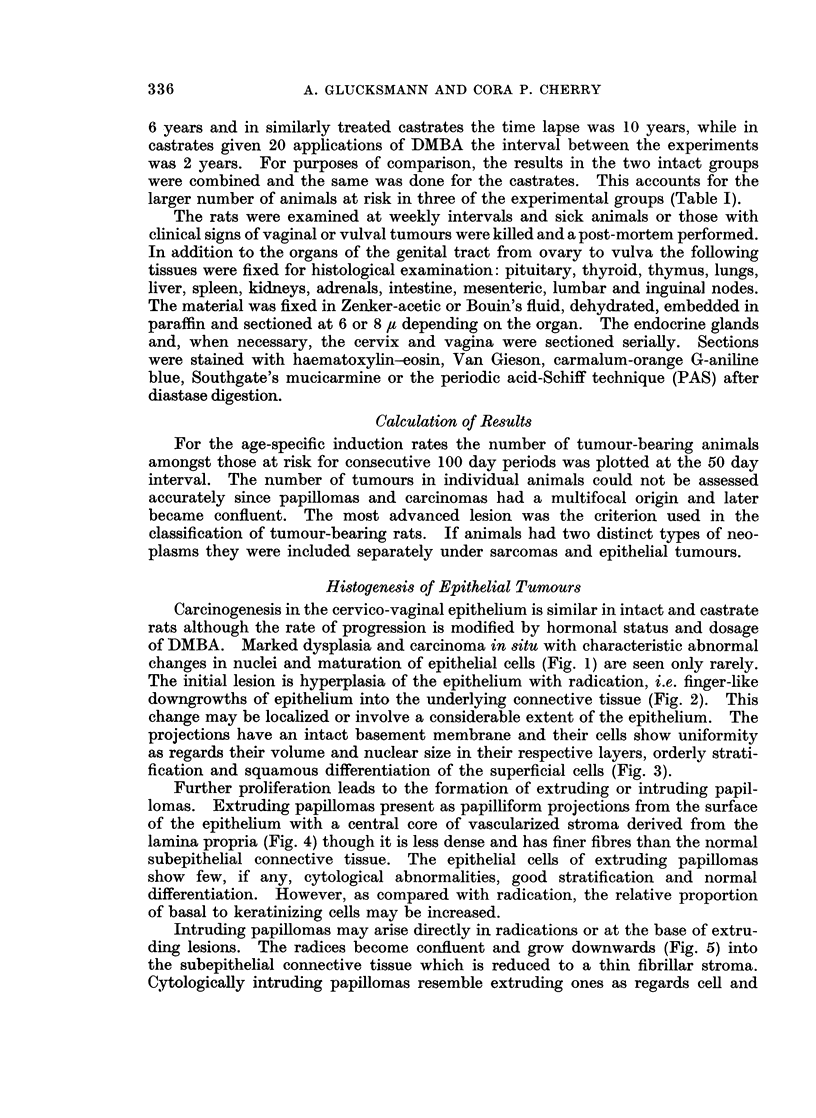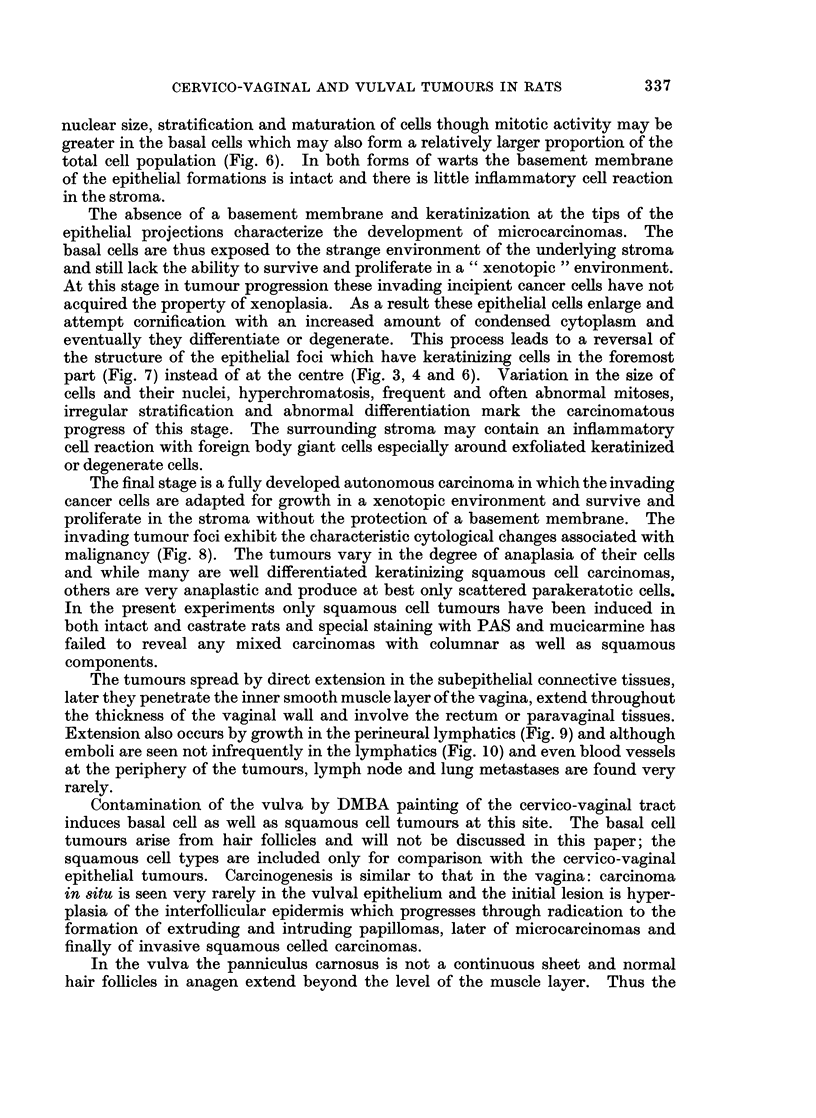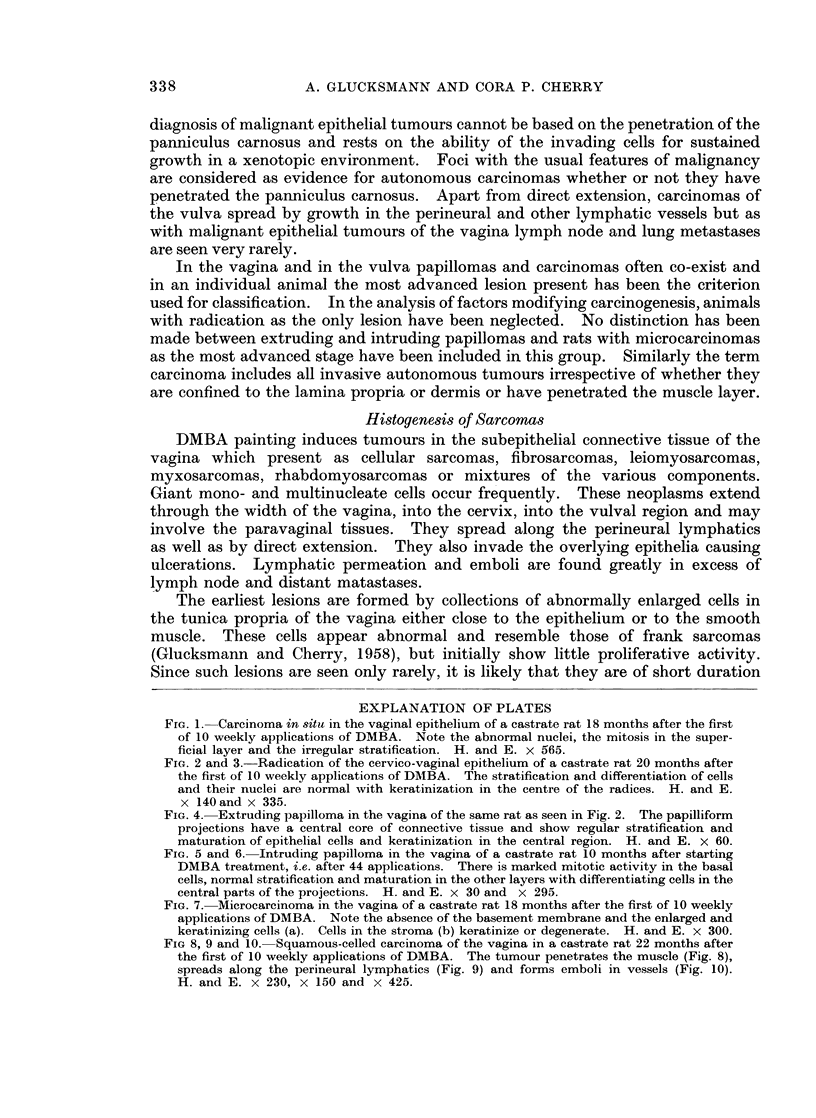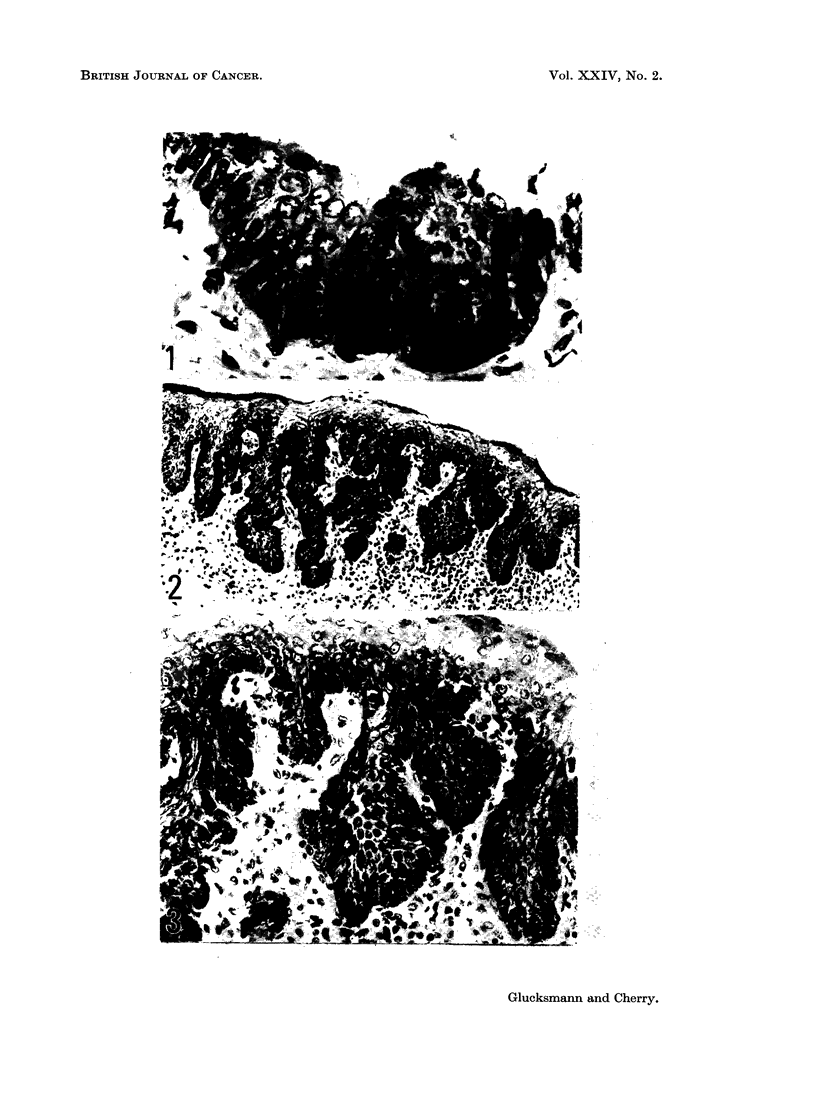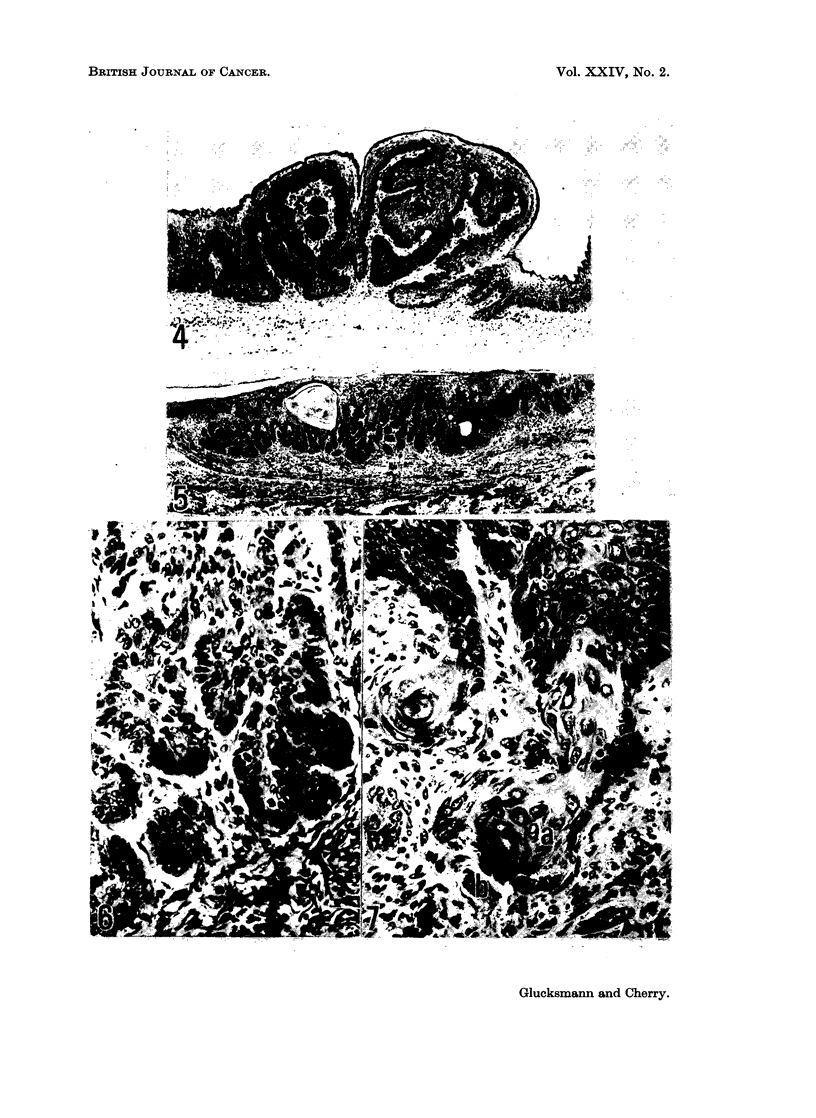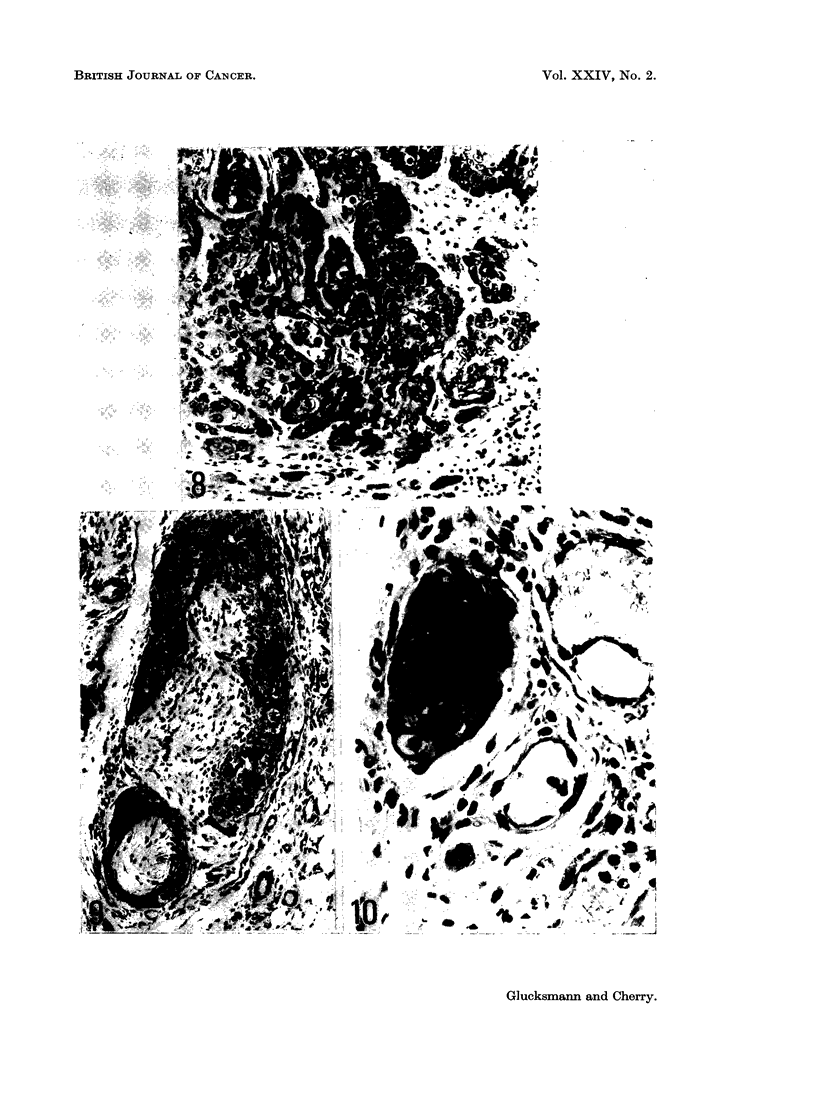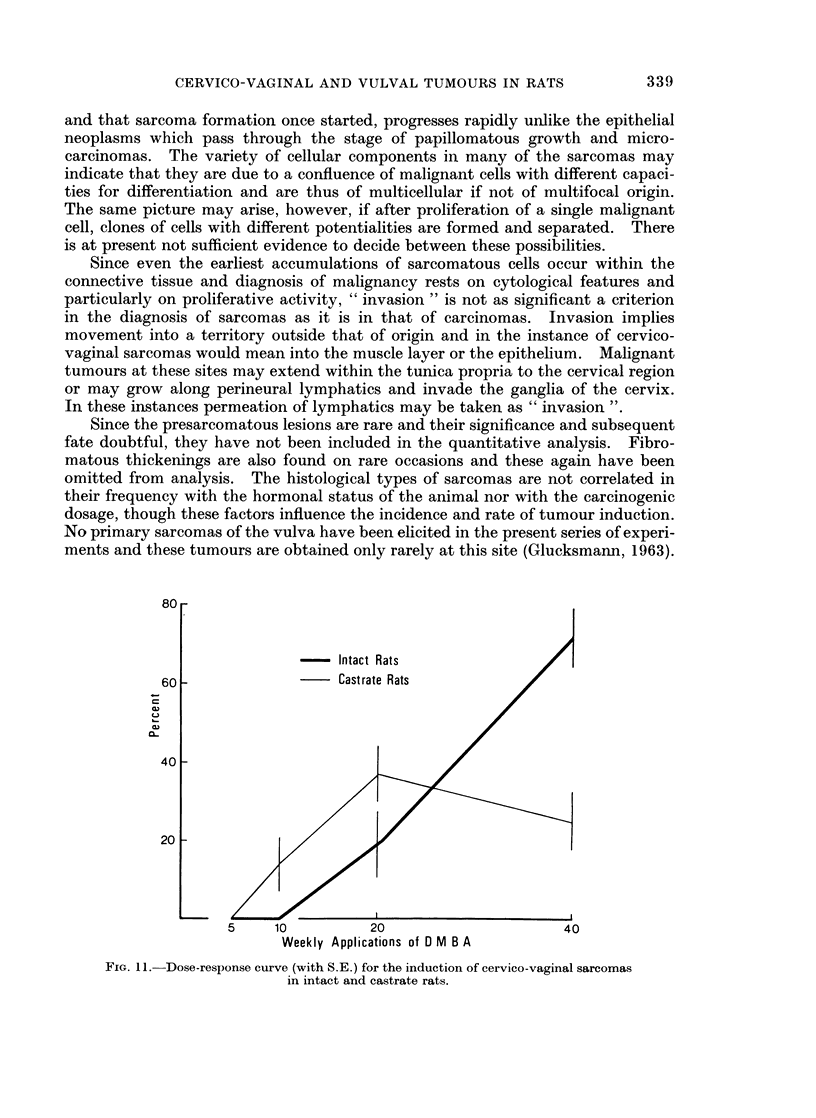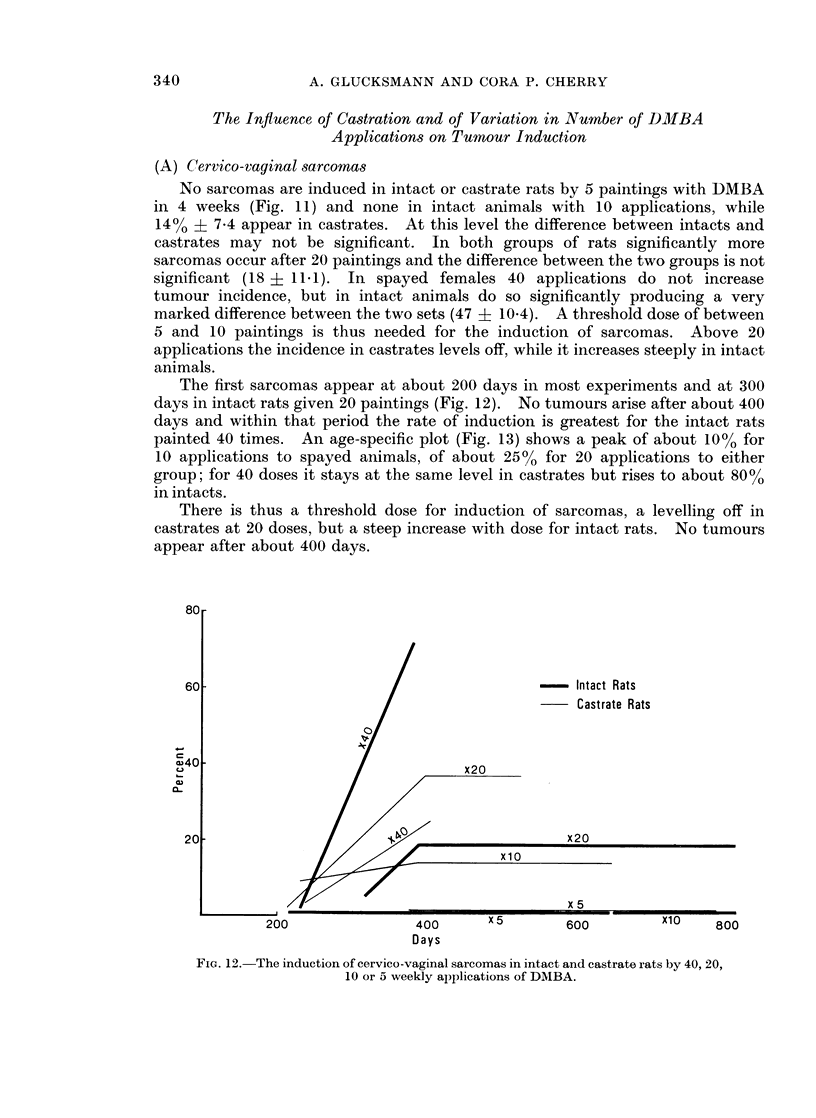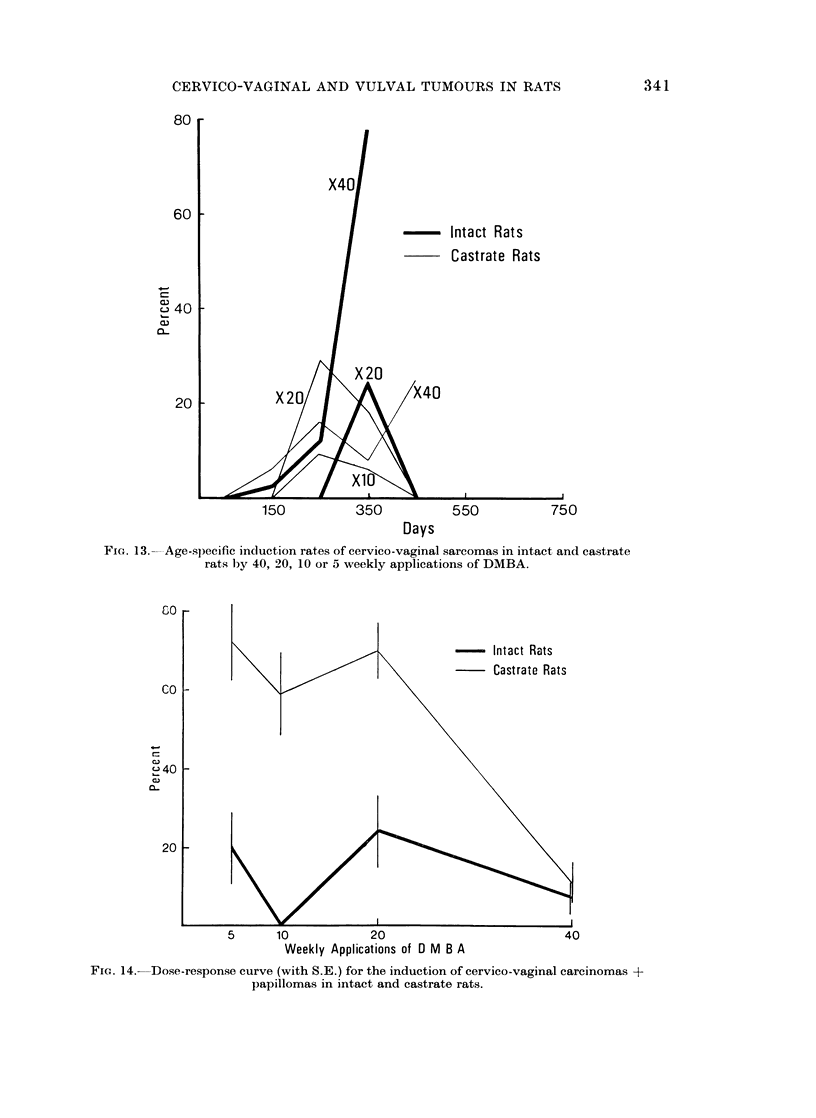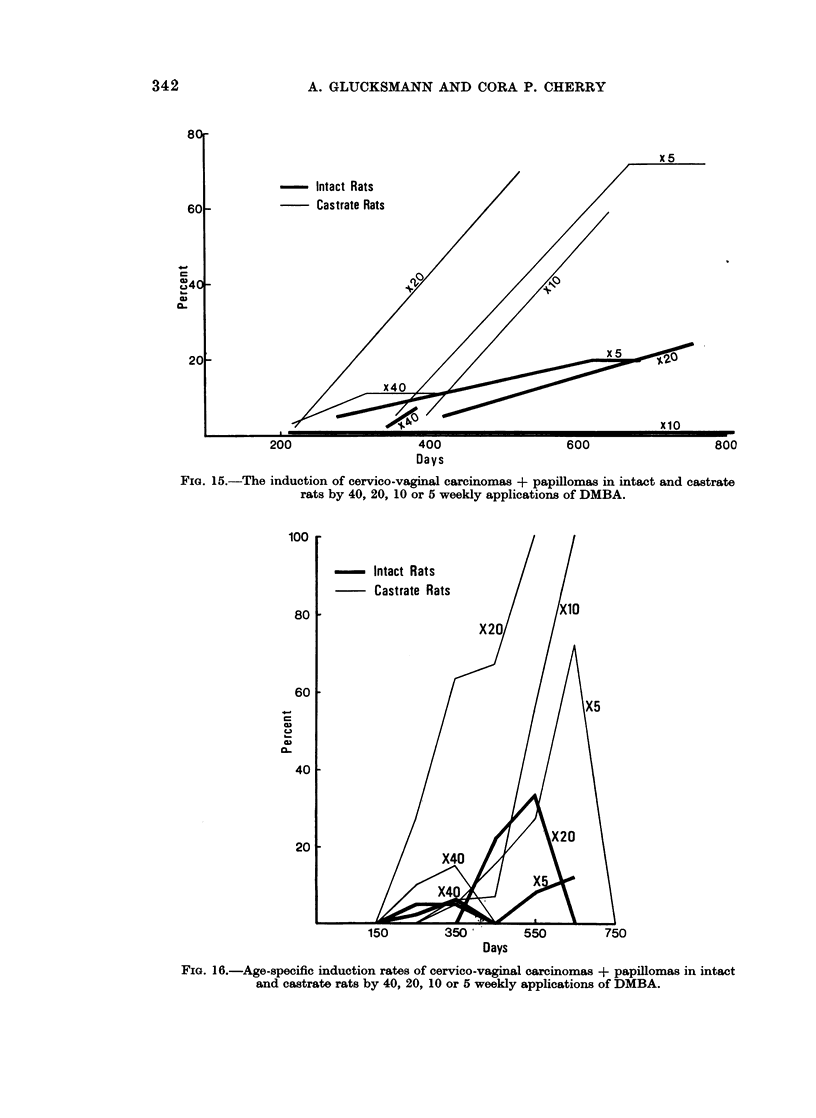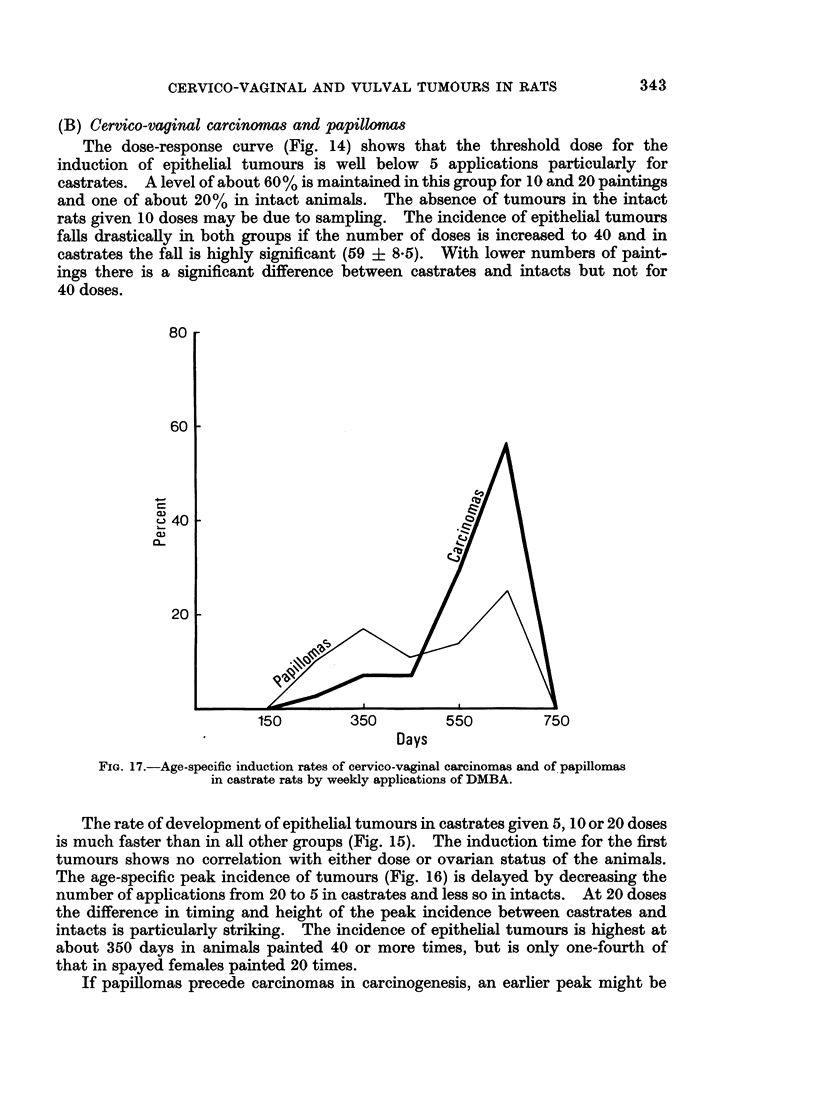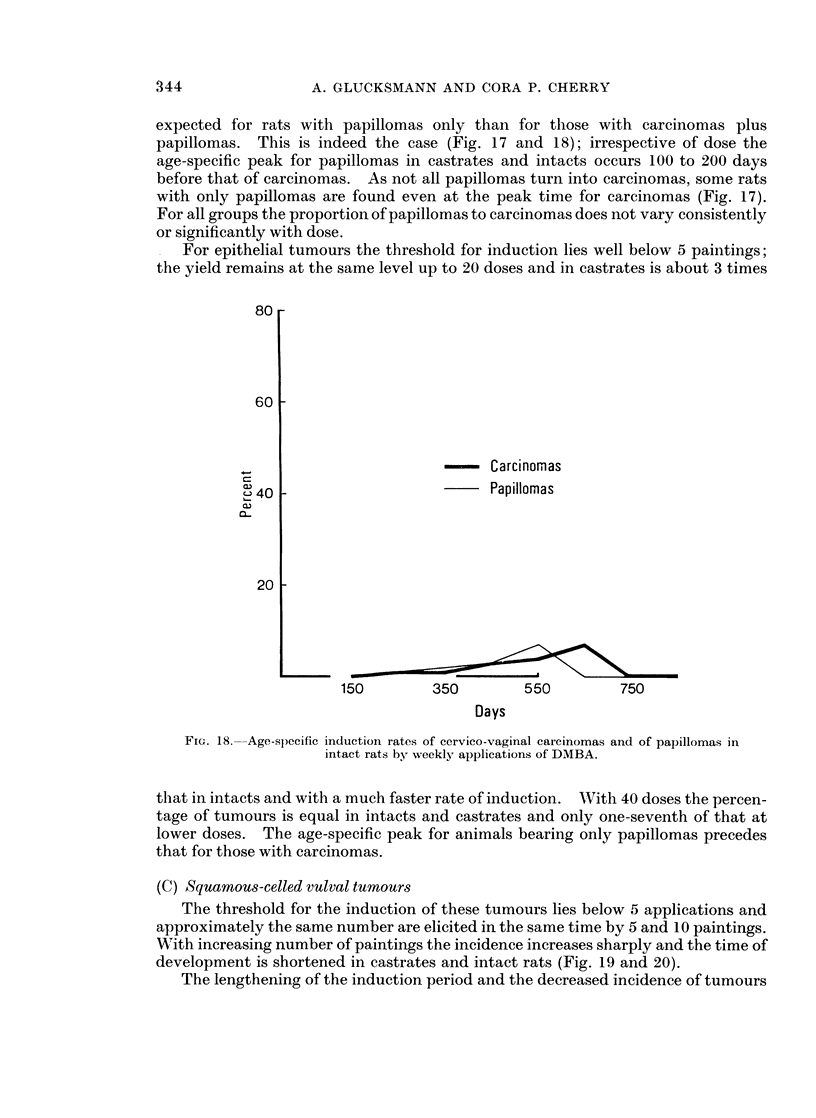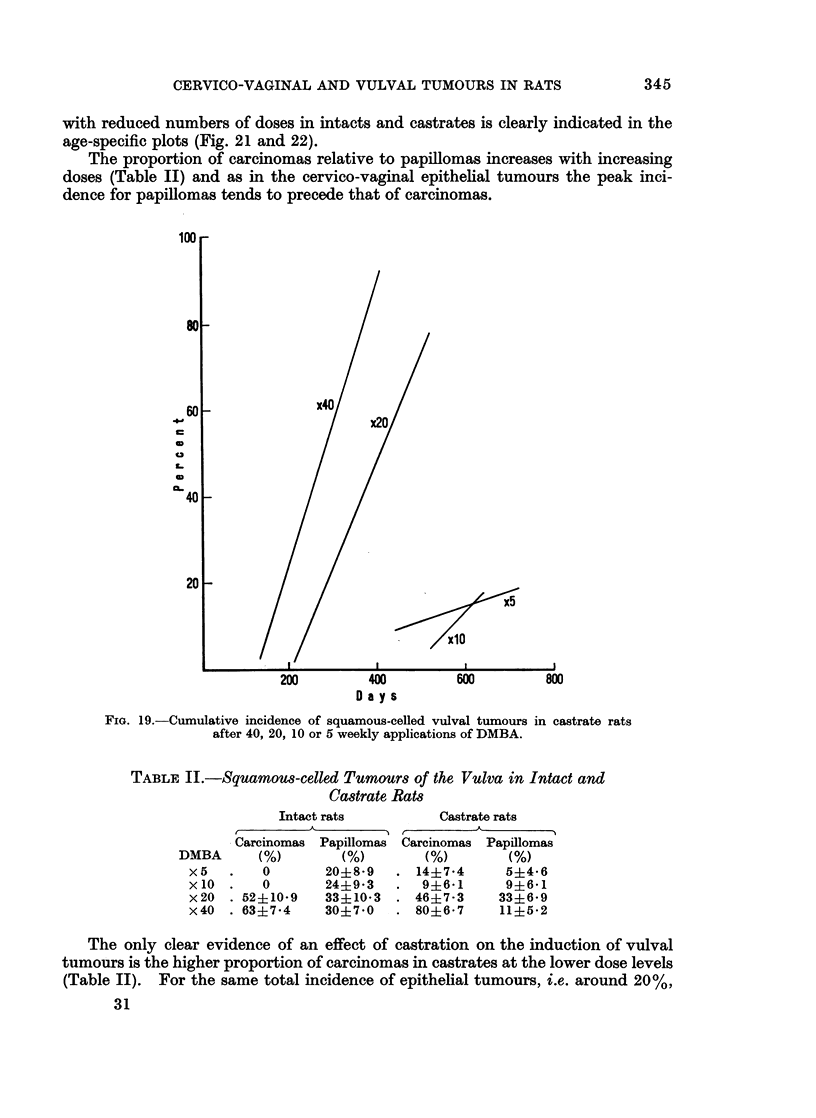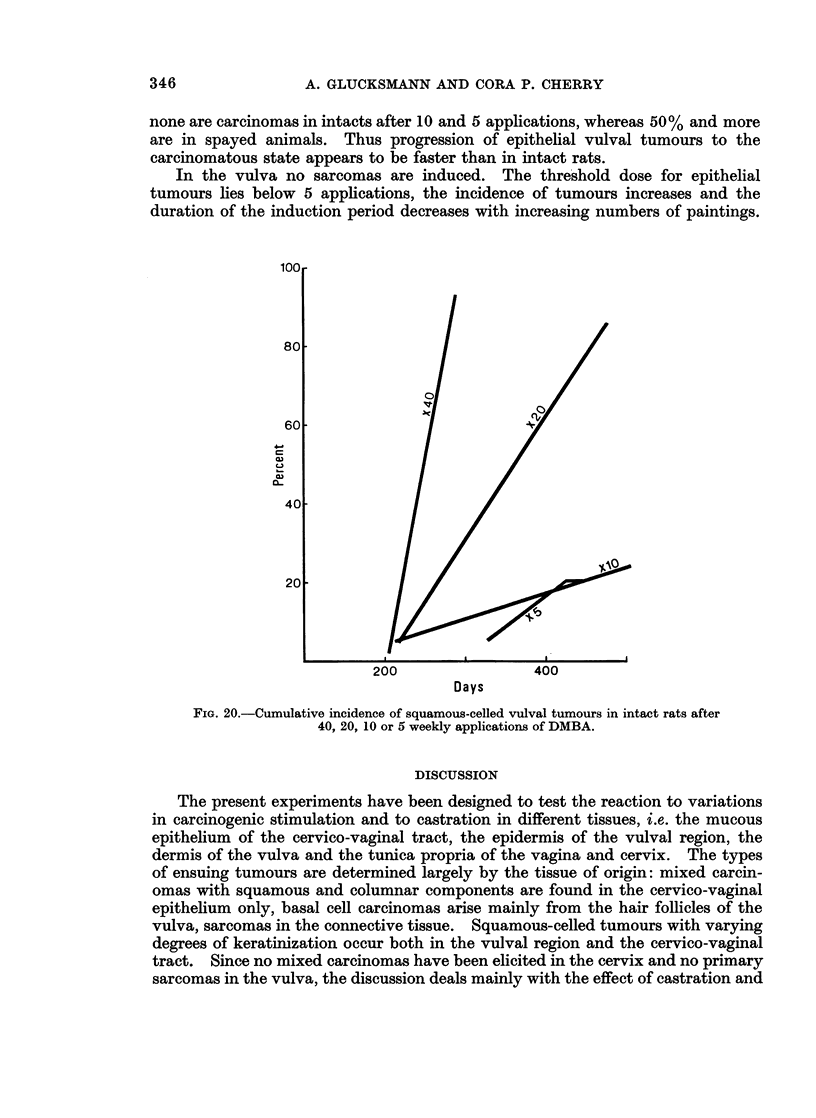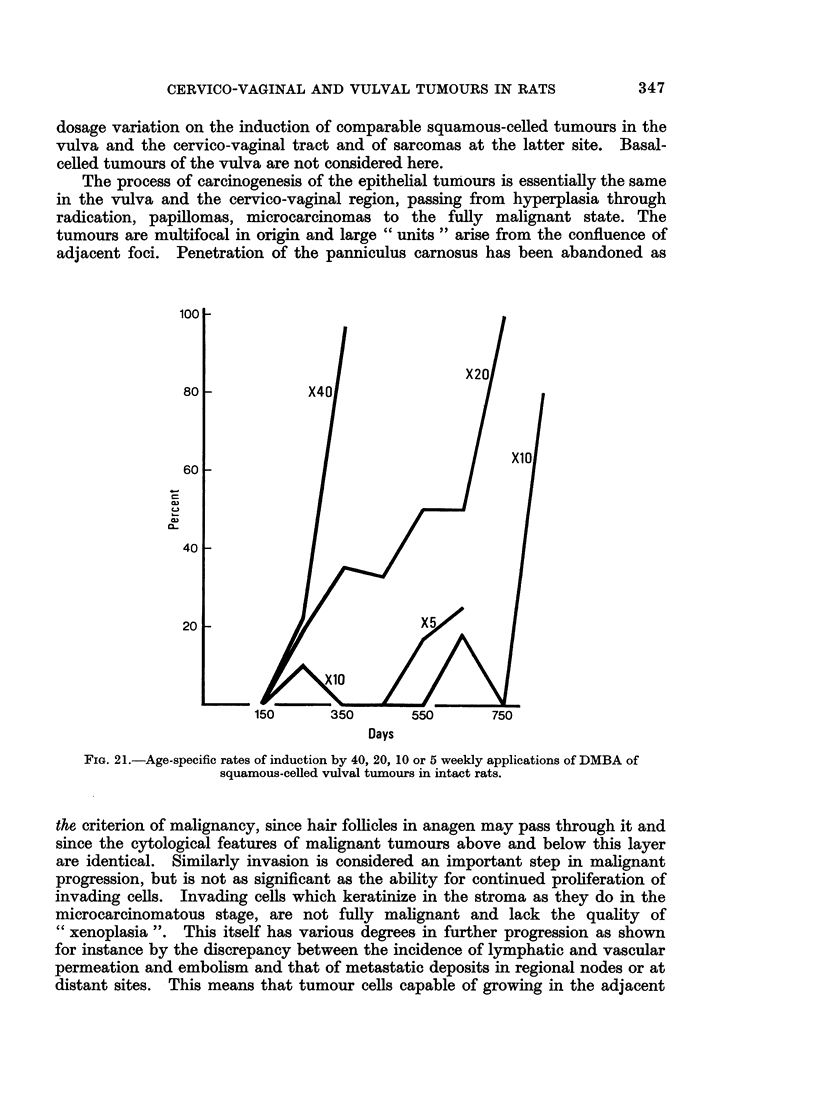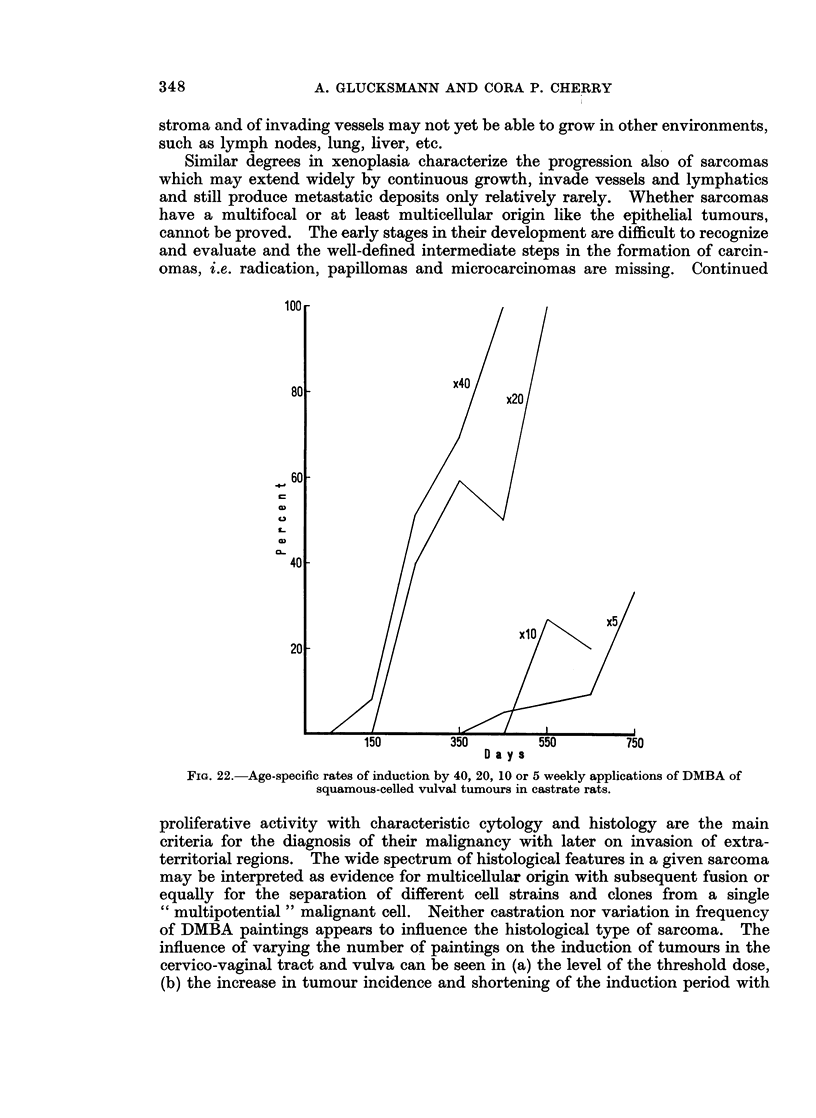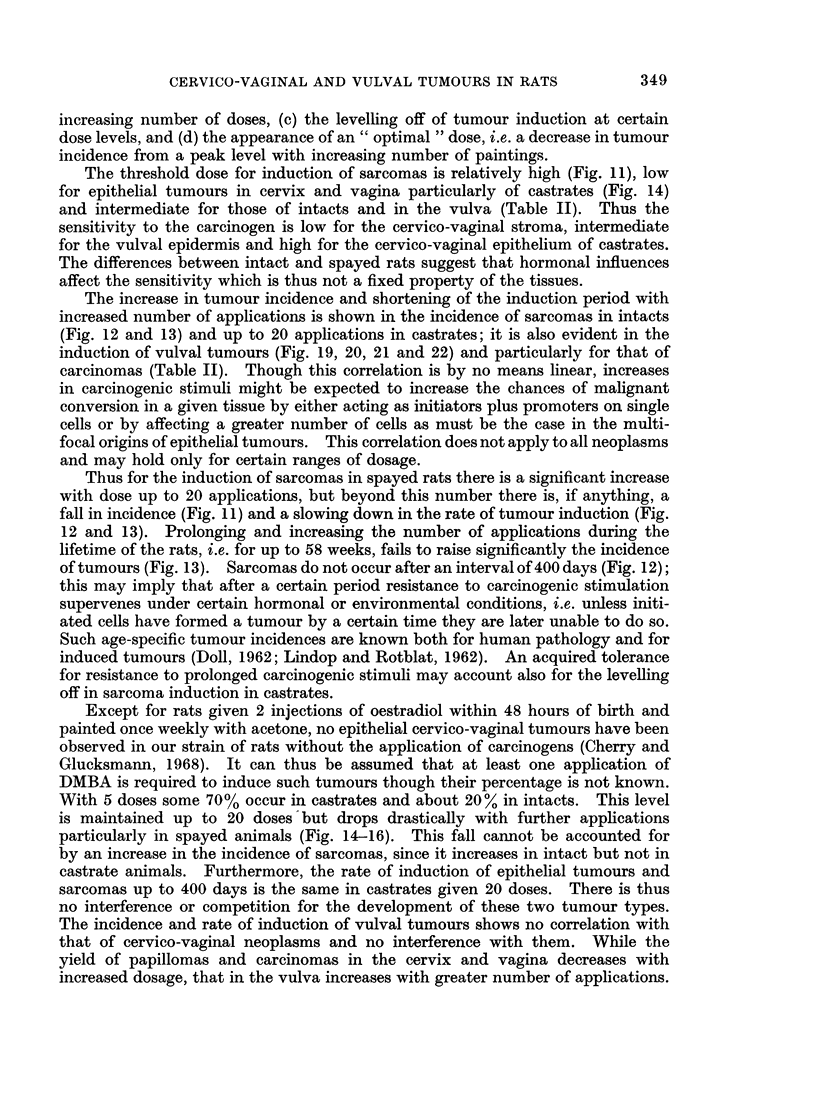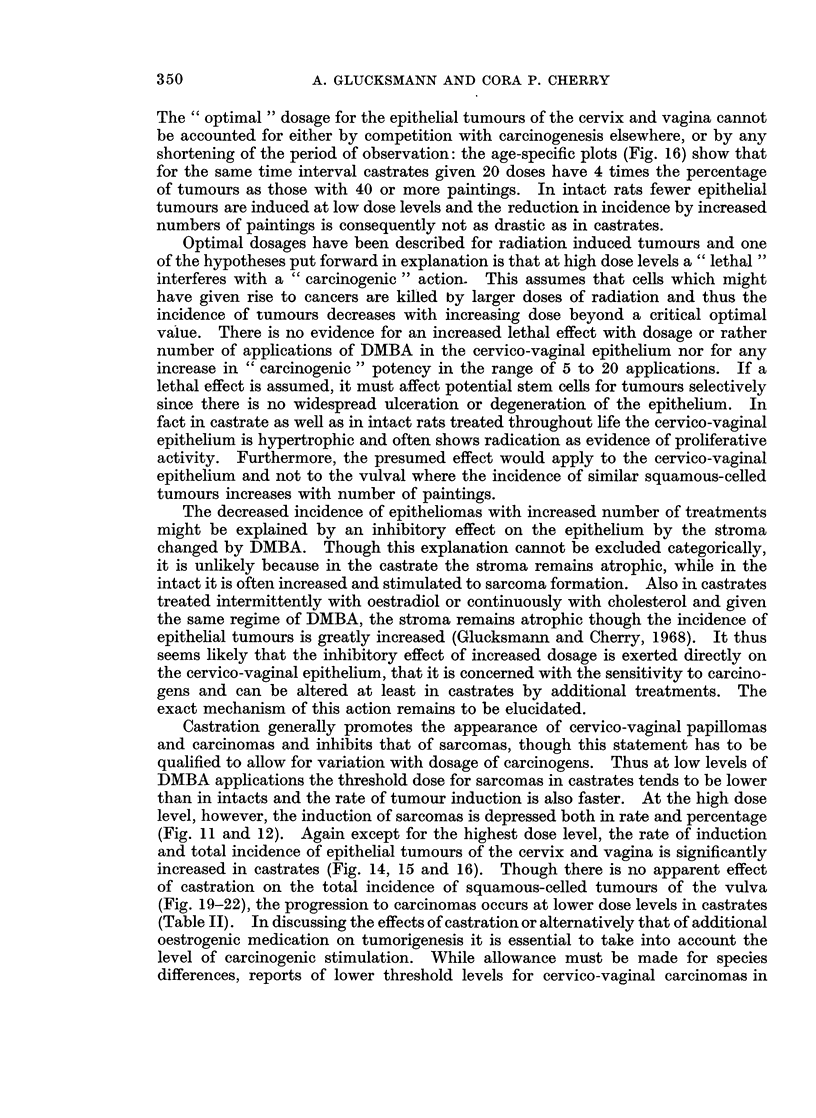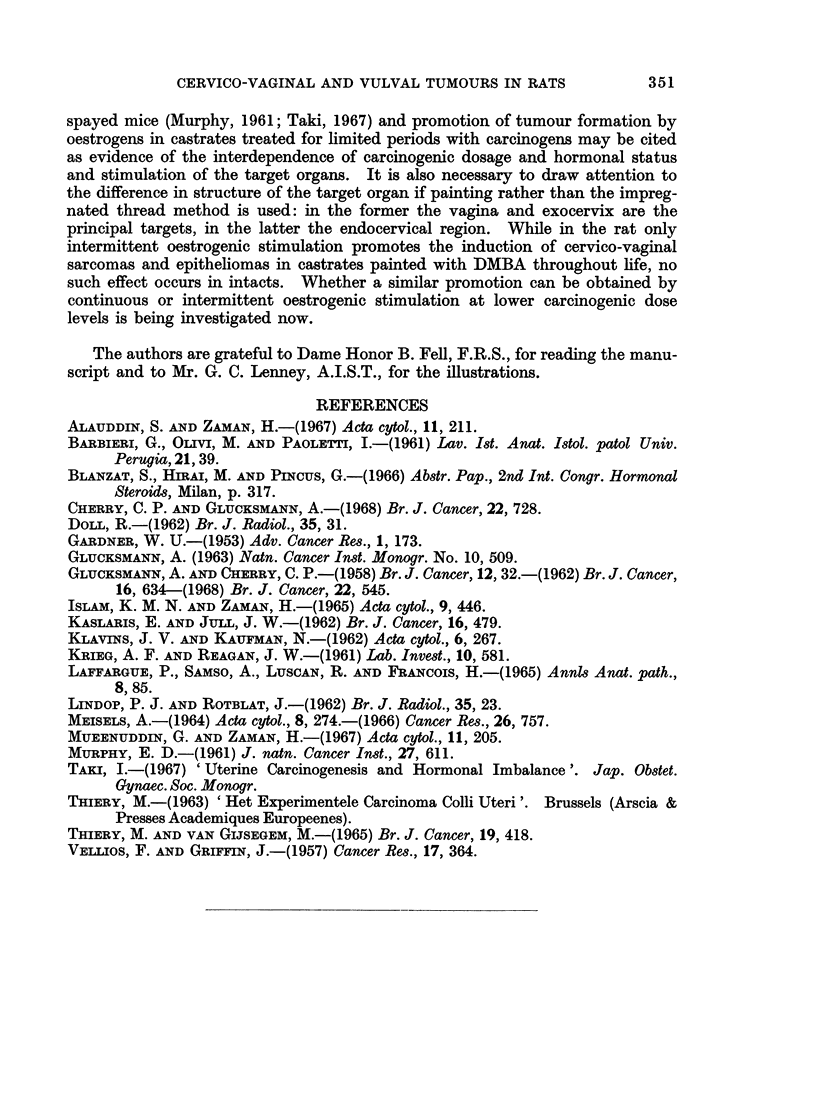# The Effect of Increased Numbers of Carcinogenic Treatments on the Induction of Cervico-Vaginal and Vulval Tumours in Intact and Castrate Rats

**DOI:** 10.1038/bjc.1970.39

**Published:** 1970-06

**Authors:** A. Glucksmann, Cora P. Cherry

## Abstract

**Images:**


### THE EFFECT OF INCREASED NUMBERS OF CARCINOGENIC TREATMENTS ON THE INDUCTION OF CERVICO-VAGINAL AND VULVAL TUMOURS IN INTACT AND CASTRATE RATS

A. GLUCKSMANN* AND CORA P. CHERRY†

*From the Strangeways Research Laboratory, Cambridge*

Received for publication January 30, 1970

**SUMMARY.—The effect of 5, 10, 20 or 40 weekly local applications of DMBA on the induction of cervico-vaginal epithelial and sarcomatous tumours and on that of squamous celled vulval neoplasms was investigated in intact and castrate rats. The threshold dose increases in the following order: epithelial cervico-vaginal tumours of castrates, followed by those in intacts and by squamous celled vulval tumours and lastly by sarcomas in castrates and intacts.**

**The incidence of sarcomas levels off at about 25% after 20 doses in spayed rats, but increases to 70% with dose in intacts. All sarcomas appear between 200 and 400 days. The incidence of vulval neoplasms increases and the duration of the induction period decreases with dose.**

**Significantly more cervico-vaginal epithelial tumours occur with 5 to 20 paintings than with further applications of DMBA. Their peak value is 60% in castrates and 20% in intacts. Castration promotes the progression of vulval papillomas to carcinomas. The sensitivity to carcinogenic stimulation is thus tissue specific and also subject to modification by hormones.**

**While epithelial tumours are multifocal and pass through well-defined intermediate stages (radication, papillomas, microcarcinomas) to full malignancy, the early stages of sarcoma formation are rarely detected and ill-defined. For epitheliomas and sarcomas " invasion " is a criterion of malignancy only if invading cells have acquired " xenoplasia ", *i.e.* the ability to grow in new environments. This capacity increases progressively and its initial lack accounts for the discrepancy between the incidence of embolism and that of metastatic deposits.**

Mice have been used extensively and rats infrequently for the induction of cervico-vaginal tumours by the local application of chemical carcinogens. Strains of mice differ in susceptibility (Kaslaris and Jull, 1962; Thiery, 1963; Thiery and van Gijsegem, 1965) and results vary with the chemical used and the technique of application. Thus for methylcholanthrene the thread method is more effective than painting of the genital tract (Murphy, 1961) while the reverse is true for benzopyrene, especially when the painting is done visually (Thiery, 1963).

There are contradictory reports on the effect of castration on experimental carcinogenesis which can be explained to some extent by differences in the period of observation. Thus in mice castration appears to enhance and accelerate the process (Krieg and Reagan, 1961; Islam and Zaman, 1965; Laffargue, Samso,

* Gibb Senior Fellow, British Empire Cancer Campaign for Research.
† Working with a grant from the British Empire Cancer Campaign for Research.

Luscan and Francois, 1965; Taki, 1967; Alauddin and Zaman, 1967; Mueenuddin and Zaman, 1967) in short term experiments with limited exposure to the carcinogen. With prolonged carcinogenic exposure Murphy (1961) and Glucksmann and Cherry (1962) found no effect of castration on tumour incidence in mice while Meisels (1966) reported an inhibitory action. The age of animals at ovariectomy is significant since castrate adult mice are less susceptible to tumour induction than those spayed when immature (Kaslaris and Jull, 1962). In rats, castration significantly reduces the incidence of cervico-vaginal sarcomas (Glucksmann and Cherry, 1958).

The effects of combining oestrogen administration with that of chemical carcinogens vary with the dose of the hormone, the length of the exposure period to the carcinogen and the species used. In mice, prolonged exposure and fairly high doses of oestrogens given to intact and castrate animals have no effect on carcinogenesis (Murphy 1961; Klavins and Kaufman, 1962; Laffargue *et al.*, 1965; Blanzat, Hirai and Pincus, 1966). Meisels (1966) reports that high doses promote tumour formation in intact mice while low or physiological levels inhibit it in both intact and castrate animals. After limited carcinogenic exposure of castrates, subsequent treatment with diethylstilboestrol pellets enhances and accelerates tumour formation and this effect is more marked with high than with low doses of hormone (Murphy, 1961). In rats, oestrogens in doses sufficient to restore to normal the castrate uterus fail to promote the induction of vaginal sarcomas in castrates and even inhibit it in intact animals. Smaller doses insufficient to counteract the uterine atrophy, increase and accelerate the formation of sarcomas and of epithelial tumours of the cervico-vaginal tract in castrates but have no effect in intacts (Glucksmann and Cherry, 1968). The different effects of oestrogens on tumour induction in mice and rats may be due to dissimilar sensitivities to this hormone in the two species and for mice it has been shown that different strains vary in their response to oestrogens (Gardner, 1953; Murphy, 1961).

The use of cholesterol as carrier for oestrogen in pellets complicates the issue since by itself it increases the formation of sarcomas and epithelial tumours in spayed rats (Glucksmann and Cherry, 1968) and in castrate mice treated and observed for only 5 weeks (Taki, 1967). Septic pyometra caused by high doses of oestrogens may promote tumour formation in mice (Gardner, 1953; Murphy, 1961) though in rats have a slightly inhibitory effect (Glucksmann and Cherry, 1968).

The histological type of induced tumour also differs in the two species. In mice most of the tumours have been carcinomas though some carcinogens have induced sarcomas (Murphy, 1961; Kaslaris and Jull, 1962; Meisels, 1964, 1966). While the majority of the carcinomas have been of the squamous cell type, some mucoepidermoid cancers have been reported (Murphy, 1961; Barbieri, Olivi and Paoletti, 1961; Thiery and van Gijsegem, 1965; Taki, 1967) and their incidence is increased by castration with or without additional treatment with progesterone (Glucksmann and Cherry, 1962). In Murphy's experiments all mixed carcinomas occur in castrate mice and none in intact animals or in castrates given additional oestrogens. Similarly Klavins and Kaufman (1962) report more highly differentiated squamous celled cancers in intact mice treated with oestrogen in combination with methylcholanthrene threads. In rats with DMBA-impregnated threads in the endocervical canal, the tumours induced are mainly carcinomas including one adenoacanthoma (Vellios and Griffin, 1957).

A carcinogen-impregnated thread inserted into the endocervical canal may be expected to induce more mixed carcinomas than painting the exocervix and vagina of mice because of the differences in histology though under certain conditions (Murphy, 1961; Glucksmann and Cherry, 1962) mucoepidermoid cancers occur after painting the cervix and vagina. The mucifying effect on the cervico-vaginal epithelium of castration and progesterone are probably responsible for the induction of mixed carcinomas.

There is a species predisposition for carcinomas in mice and for sarcomas in rats following intravaginal painting. However, sarcomas occur in some mice and in intact and castrate rats epithelial tumours are induced by additional treatment with testosterone (Glucksmann and Cherry, 1968). Low doses of stilboestrol or cholesterol pellets in castrates increase the incidence of sarcomas and of epitheliomas.

In rats painted weekly with carcinogens the induction period for tumours is very long and it is feasible that some of the carcinogenic insults are wasted. Differences in carcinogenic dosage may account for some of the conflicting results obtained with castration and for the histological type of the induced tumours, *i.e.* whether epithelial or sarcomatous. In the present experiments the effect of 5, 10, 20 and nominally 40 weekly intravaginal applications of DMBA have been investigated in intact as well as castrate rats.

### MATERIALS AND METHODS

Hooded rats of the Lister strain random bred within a closed colony since 1940 were used for the experiments which extended over a period from 1955 to 1967. The rats were housed 7 to a cage and given water and food pellets of MRC–diet 86 *ad libitum.* Only animals surviving for at least 120 days after starting the experiment were considered at risk. The number of animals in the various treatment groups are given in Table I.

Bilateral ovariectomy was performed with a dorsal approach under ether anaesthesia on rats aged 4 to 5 weeks. Carcinogenic treatment with a 1% solution in acetone of 9,10-dimethyl-1,2-benzanthracene (DMBA, Koch–Light Ltd.) was started when intact and castrate animals were 2 months old. The vagina was stretched open by dorsal flexion of the tail and, by means of a cotton wool swab mounted on a thin wire rod, the solution was distributed by a rotary motion over the cervix, vagina and introitus at weekly intervals either for the life span of the animals (with an average of 40 applications) or restricted to 5, 10 or 20 times (Table I).

TABLE I.—*Number of Animals at risk for Different Numbers of Weekly Applications of DMBA*

| DMBA | Intact rats | Castrate rats |
|---|---|---|
| ×5 | 20 | 22 |
| ×10 | 20 | 22 |
| ×20 | 21 | 46 |
| ×40 | 43 | 36 |

Some of the experiments were repeated after intervals of 2 to 10 years, and gave almost identical figures for the induction of tumours. In intact rats treated for an average of 40 weeks the repeat experiment was done after an interval of

6 years and in similarly treated castrates the time lapse was 10 years, while in castrates given 20 applications of DMBA the interval between the experiments was 2 years. For purposes of comparison, the results in the two intact groups were combined and the same was done for the castrates. This accounts for the larger number of animals at risk in three of the experimental groups (Table I).

The rats were examined at weekly intervals and sick animals or those with clinical signs of vaginal or vulval tumours were killed and a post-mortem performed. In addition to the organs of the genital tract from ovary to vulva the following tissues were fixed for histological examination: pituitary, thyroid, thymus, lungs, liver, spleen, kidneys, adrenals, intestine, mesenteric, lumbar and inguinal nodes. The material was fixed in Zenker-acetic or Bouin's fluid, dehydrated, embedded in paraffin and sectioned at 6 or 8 $\mu$ depending on the organ. The endocrine glands and, when necessary, the cervix and vagina were sectioned serially. Sections were stained with haematoxylin–eosin, Van Gieson, carmalum-orange G-aniline blue, Southgate's mucicarmine or the periodic acid-Schiff technique (PAS) after diastase digestion.

### *Calculation of Results*

For the age-specific induction rates the number of tumour-bearing animals amongst those at risk for consecutive 100 day periods was plotted at the 50 day interval. The number of tumours in individual animals could not be assessed accurately since papillomas and carcinomas had a multifocal origin and later became confluent. The most advanced lesion was the criterion used in the classification of tumour-bearing rats. If animals had two distinct types of neoplasms they were included separately under sarcomas and epithelial tumours.

### *Histogenesis of Epithelial Tumours*

Carcinogenesis in the cervico-vaginal epithelium is similar in intact and castrate rats although the rate of progression is modified by hormonal status and dosage of DMBA. Marked dysplasia and carcinoma *in situ* with characteristic abnormal changes in nuclei and maturation of epithelial cells (Fig. 1) are seen only rarely. The initial lesion is hyperplasia of the epithelium with radication, *i.e.* finger-like downgrowths of epithelium into the underlying connective tissue (Fig. 2). This change may be localized or involve a considerable extent of the epithelium. The projections have an intact basement membrane and their cells show uniformity as regards their volume and nuclear size in their respective layers, orderly stratification and squamous differentiation of the superficial cells (Fig. 3).

Further proliferation leads to the formation of extruding or intruding papillomas. Extruding papillomas present as papilliform projections from the surface of the epithelium with a central core of vascularized stroma derived from the lamina propria (Fig. 4) though it is less dense and has finer fibres than the normal subepithelial connective tissue. The epithelial cells of extruding papillomas show few, if any, cytological abnormalities, good stratification and normal differentiation. However, as compared with radication, the relative proportion of basal to keratinizing cells may be increased.

Intruding papillomas may arise directly in radications or at the base of extruding lesions. The radices become confluent and grow downwards (Fig. 5) into the subepithelial connective tissue which is reduced to a thin fibrillar stroma. Cytologically intruding papillomas resemble extruding ones as regards cell and nuclear size, stratification and maturation of cells though mitotic activity may be greater in the basal cells which may also form a relatively larger proportion of the total cell population (Fig. 6). In both forms of warts the basement membrane of the epithelial formations is intact and there is little inflammatory cell reaction in the stroma.

The absence of a basement membrane and keratinization at the tips of the epithelial projections characterize the development of microcarcinomas. The basal cells are thus exposed to the strange environment of the underlying stroma and still lack the ability to survive and proliferate in a " xenotopic " environment. At this stage in tumour progression these invading incipient cancer cells have not acquired the property of xenoplasia. As a result these epithelial cells enlarge and attempt cornification with an increased amount of condensed cytoplasm and eventually they differentiate or degenerate. This process leads to a reversal of the structure of the epithelial foci which have keratinizing cells in the foremost part (Fig. 7) instead of at the centre (Fig. 3, 4 and 6). Variation in the size of cells and their nuclei, hyperchromatosis, frequent and often abnormal mitoses, irregular stratification and abnormal differentiation mark the carcinomatous progress of this stage. The surrounding stroma may contain an inflammatory cell reaction with foreign body giant cells especially around exfoliated keratinized or degenerate cells.

The final stage is a fully developed autonomous carcinoma in which the invading cancer cells are adapted for growth in a xenotopic environment and survive and proliferate in the stroma without the protection of a basement membrane. The invading tumour foci exhibit the characteristic cytological changes associated with malignancy (Fig. 8). The tumours vary in the degree of anaplasia of their cells and while many are well differentiated keratinizing squamous cell carcinomas, others are very anaplastic and produce at best only scattered parakeratotic cells. In the present experiments only squamous cell tumours have been induced in both intact and castrate rats and special staining with PAS and mucicarmine has failed to reveal any mixed carcinomas with columnar as well as squamous components.

The tumours spread by direct extension in the subepithelial connective tissues, later they penetrate the inner smooth muscle layer of the vagina, extend throughout the thickness of the vaginal wall and involve the rectum or paravaginal tissues. Extension also occurs by growth in the perineural lymphatics (Fig. 9) and although emboli are seen not infrequently in the lymphatics (Fig. 10) and even blood vessels at the periphery of the tumours, lymph node and lung metastases are found very rarely.

Contamination of the vulva by DMBA painting of the cervico-vaginal tract induces basal cell as well as squamous cell tumours at this site. The basal cell tumours arise from hair follicles and will not be discussed in this paper; the squamous cell types are included only for comparison with the cervico-vaginal epithelial tumours. Carcinogenesis is similar to that in the vagina: carcinoma *in situ* is seen very rarely in the vulval epithelium and the initial lesion is hyperplasia of the interfollicular epidermis which progresses through radication to the formation of extruding and intruding papillomas, later of microcarcinomas and finally of invasive squamous celled carcinomas.

In the vulva the panniculus carnosus is not a continuous sheet and normal hair follicles in anagen extend beyond the level of the muscle layer. Thus the diagnosis of malignant epithelial tumours cannot be based on the penetration of the panniculus carnosus and rests on the ability of the invading cells for sustained growth in a xenotopic environment. Foci with the usual features of malignancy are considered as evidence for autonomous carcinomas whether or not they have penetrated the panniculus carnosus. Apart from direct extension, carcinomas of the vulva spread by growth in the perineural and other lymphatic vessels but as with malignant epithelial tumours of the vagina lymph node and lung metastases are seen very rarely.

In the vagina and in the vulva papillomas and carcinomas often co-exist and in an individual animal the most advanced lesion present has been the criterion used for classification. In the analysis of factors modifying carcinogenesis, animals with radication as the only lesion have been neglected. No distinction has been made between extruding and intruding papillomas and rats with microcarcinomas as the most advanced stage have been included in this group. Similarly the term carcinoma includes all invasive autonomous tumours irrespective of whether they are confined to the lamina propria or dermis or have penetrated the muscle layer.

*Histogenesis of Sarcomas*

DMBA painting induces tumours in the subepithelial connective tissue of the vagina which present as cellular sarcomas, fibrosarcomas, leiomyosarcomas, myxosarcomas, rhabdomyosarcomas or mixtures of the various components. Giant mono- and multinucleate cells occur frequently. These neoplasms extend through the width of the vagina, into the cervix, into the vulval region and may involve the paravaginal tissues. They spread along the perineural lymphatics as well as by direct extension. They also invade the overlying epithelia causing ulcerations. Lymphatic permeation and emboli are found greatly in excess of lymph node and distant matastases.

The earliest lesions are formed by collections of abnormally enlarged cells in the tunica propria of the vagina either close to the epithelium or to the smooth muscle. These cells appear abnormal and resemble those of frank sarcomas (Glucksmann and Cherry, 1958), but initially show little proliferative activity. Since such lesions are seen only rarely, it is likely that they are of short duration

EXPLANATION OF PLATES

FIG. 1.—Carcinoma *in situ* in the vaginal epithelium of a castrate rat 18 months after the first of 10 weekly applications of DMBA. Note the abnormal nuclei, the mitosis in the superficial layer and the irregular stratification. H. and E. × 565.

FIG. 2 and 3.—Radication of the cervico-vaginal epithelium of a castrate rat 20 months after the first of 10 weekly applications of DMBA. The stratification and differentiation of cells and their nuclei are normal with keratinization in the centre of the radices. H. and E. × 140 and × 335.

FIG. 4.—Extruding papilloma in the vagina of the same rat as seen in Fig. 2. The papilliform projections have a central core of connective tissue and show regular stratification and maturation of epithelial cells and keratinization in the central region. H. and E. × 60.

FIG. 5 and 6.—Intruding papilloma in the vagina of a castrate rat 10 months after starting DMBA treatment, *i.e.* after 44 applications. There is marked mitotic activity in the basal cells, normal stratification and maturation in the other layers with differentiating cells in the central parts of the projections. H. and E. × 30 and × 295.

FIG. 7.—Microcarcinoma in the vagina of a castrate rat 18 months after the first of 10 weekly applications of DMBA. Note the absence of the basement membrane and the enlarged and keratinizing cells (a). Cells in the stroma (b) keratinize or degenerate. H. and E. × 300.

FIG 8, 9 and 10.—Squamous-celled carcinoma of the vagina in a castrate rat 22 months after the first of 10 weekly applications of DMBA. The tumour penetrates the muscle (Fig. 8), spreads along the perineural lymphatics (Fig. 9) and forms emboli in vessels (Fig. 10). H. and E. × 230, × 150 and × 425.

Glucksmann and Cherry.

Glucksmann and Cherry.

Glucksmann and Cherry.

and that sarcoma formation once started, progresses rapidly unlike the epithelial neoplasms which pass through the stage of papillomatous growth and micro-carcinomas. The variety of cellular components in many of the sarcomas may indicate that they are due to a confluence of malignant cells with different capacities for differentiation and are thus of multicellular if not of multifocal origin. The same picture may arise, however, if after proliferation of a single malignant cell, clones of cells with different potentialities are formed and separated. There is at present not sufficient evidence to decide between these possibilities.

Since even the earliest accumulations of sarcomatous cells occur within the connective tissue and diagnosis of malignancy rests on cytological features and particularly on proliferative activity, " invasion " is not as significant a criterion in the diagnosis of sarcomas as it is in that of carcinomas. Invasion implies movement into a territory outside that of origin and in the instance of cervico-vaginal sarcomas would mean into the muscle layer or the epithelium. Malignant tumours at these sites may extend within the tunica propria to the cervical region or may grow along perineural lymphatics and invade the ganglia of the cervix. In these instances permeation of lymphatics may be taken as " invasion ".

Since the presarcomatous lesions are rare and their significance and subsequent fate doubtful, they have not been included in the quantitative analysis. Fibromatous thickenings are also found on rare occasions and these again have been omitted from analysis. The histological types of sarcomas are not correlated in their frequency with the hormonal status of the animal nor with the carcinogenic dosage, though these factors influence the incidence and rate of tumour induction. No primary sarcomas of the vulva have been elicited in the present series of experiments and these tumours are obtained only rarely at this site (Glucksmann, 1963).

FIG. 11.—Dose-response curve (with S.E.) for the induction of cervico-vaginal sarcomas in intact and castrate rats.

### *The Influence of Castration and of Variation in Number of DMBA Applications on Tumour Induction*

(A) *Cervico-vaginal sarcomas*

No sarcomas are induced in intact or castrate rats by 5 paintings with DMBA in 4 weeks (Fig. 11) and none in intact animals with 10 applications, while 14% ± 7·4 appear in castrates. At this level the difference between intacts and castrates may not be significant. In both groups of rats significantly more sarcomas occur after 20 paintings and the difference between the two groups is not significant (18 ± 11·1). In spayed females 40 applications do not increase tumour incidence, but in intact animals do so significantly producing a very marked difference between the two sets (47 ± 10·4). A threshold dose of between 5 and 10 paintings is thus needed for the induction of sarcomas. Above 20 applications the incidence in castrates levels off, while it increases steeply in intact animals.

The first sarcomas appear at about 200 days in most experiments and at 300 days in intact rats given 20 paintings (Fig. 12). No tumours arise after about 400 days and within that period the rate of induction is greatest for the intact rats painted 40 times. An age-specific plot (Fig. 13) shows a peak of about 10% for 10 applications to spayed animals, of about 25% for 20 applications to either group; for 40 doses it stays at the same level in castrates but rises to about 80% in intacts.

There is thus a threshold dose for induction of sarcomas, a levelling off in castrates at 20 doses, but a steep increase with dose for intact rats. No tumours appear after about 400 days.

Fig. 12.—The induction of cervico-vaginal sarcomas in intact and castrate rats by 40, 20, 10 or 5 weekly applications of DMBA.

FIG. 13.—Age-specific induction rates of cervico-vaginal sarcomas in intact and castrate rats by 40, 20, 10 or 5 weekly applications of DMBA.

FIG. 14.—Dose-response curve (with S.E.) for the induction of cervico-vaginal carcinomas + papillomas in intact and castrate rats.

FIG. 15.—The induction of cervico-vaginal carcinomas + papillomas in intact and castrate rats by 40, 20, 10 or 5 weekly applications of DMBA.

FIG. 16.—Age-specific induction rates of cervico-vaginal carcinomas + papillomas in intact and castrate rats by 40, 20, 10 or 5 weekly applications of DMBA.

(B) *Cervico-vaginal carcinomas and papillomas*

The dose-response curve (Fig. 14) shows that the threshold dose for the induction of epithelial tumours is well below 5 applications particularly for castrates. A level of about 60% is maintained in this group for 10 and 20 paintings and one of about 20% in intact animals. The absence of tumours in the intact rats given 10 doses may be due to sampling. The incidence of epithelial tumours falls drastically in both groups if the number of doses is increased to 40 and in castrates the fall is highly significant (59 ± 8·5). With lower numbers of paintings there is a significant difference between castrates and intacts but not for 40 doses.

FIG. 17.—Age-specific induction rates of cervico-vaginal carcinomas and of papillomas in castrate rats by weekly applications of DMBA.

The rate of development of epithelial tumours in castrates given 5, 10 or 20 doses is much faster than in all other groups (Fig. 15). The induction time for the first tumours shows no correlation with either dose or ovarian status of the animals. The age-specific peak incidence of tumours (Fig. 16) is delayed by decreasing the number of applications from 20 to 5 in castrates and less so in intacts. At 20 doses the difference in timing and height of the peak incidence between castrates and intacts is particularly striking. The incidence of epithelial tumours is highest at about 350 days in animals painted 40 or more times, but is only one-fourth of that in spayed females painted 20 times.

If papillomas precede carcinomas in carcinogenesis, an earlier peak might be expected for rats with papillomas only than for those with carcinomas plus papillomas. This is indeed the case (Fig. 17 and 18); irrespective of dose the age-specific peak for papillomas in castrates and intacts occurs 100 to 200 days before that of carcinomas. As not all papillomas turn into carcinomas, some rats with only papillomas are found even at the peak time for carcinomas (Fig. 17). For all groups the proportion of papillomas to carcinomas does not vary consistently or significantly with dose.

For epithelial tumours the threshold for induction lies well below 5 paintings; the yield remains at the same level up to 20 doses and in castrates is about 3 times

FIG. 18.—Age-specific induction rates of cervico-vaginal carcinomas and of papillomas in intact rats by weekly applications of DMBA.

that in intacts and with a much faster rate of induction. With 40 doses the percentage of tumours is equal in intacts and castrates and only one-seventh of that at lower doses. The age-specific peak for animals bearing only papillomas precedes that for those with carcinomas.

(C) *Squamous-celled vulval tumours*

The threshold for the induction of these tumours lies below 5 applications and approximately the same number are elicited in the same time by 5 and 10 paintings. With increasing number of paintings the incidence increases sharply and the time of development is shortened in castrates and intact rats (Fig. 19 and 20).

The lengthening of the induction period and the decreased incidence of tumours with reduced numbers of doses in intacts and castrates is clearly indicated in the age-specific plots (Fig. 21 and 22).

The proportion of carcinomas relative to papillomas increases with increasing doses (Table II) and as in the cervico-vaginal epithelial tumours the peak incidence for papillomas tends to precede that of carcinomas.

FIG. 19.—Cumulative incidence of squamous-celled vulval tumours in castrate rats after 40, 20, 10 or 5 weekly applications of DMBA.

TABLE II.—*Squamous-celled Tumours of the Vulva in Intact and Castrate Rats*

| | Intact rats | | Castrate rats | |
|---|---|---|---|---|
| DMBA | Carcinomas (%) | Papillomas (%) | Carcinomas (%) | Papillomas (%) |
| ×5 | 0 | 20±8·9 | 14±7·4 | 5±4·6 |
| ×10 | 0 | 24±9·3 | 9±6·1 | 9±6·1 |
| ×20 | 52±10·9 | 33±10·3 | 46±7·3 | 33±6·9 |
| ×40 | 63±7·4 | 30±7·0 | 80±6·7 | 11±5·2 |

The only clear evidence of an effect of castration on the induction of vulval tumours is the higher proportion of carcinomas in castrates at the lower dose levels (Table II). For the same total incidence of epithelial tumours, *i.e.* around 20%, none are carcinomas in intacts after 10 and 5 applications, whereas 50% and more are in spayed animals. Thus progression of epithelial vulval tumours to the carcinomatous state appears to be faster than in intact rats.

In the vulva no sarcomas are induced. The threshold dose for epithelial tumours lies below 5 applications, the incidence of tumours increases and the duration of the induction period decreases with increasing numbers of paintings.

FIG. 20.—Cumulative incidence of squamous-celled vulval tumours in intact rats after 40, 20, 10 or 5 weekly applications of DMBA.

### DISCUSSION

The present experiments have been designed to test the reaction to variations in carcinogenic stimulation and to castration in different tissues, *i.e.* the mucous epithelium of the cervico-vaginal tract, the epidermis of the vulval region, the dermis of the vulva and the tunica propria of the vagina and cervix. The types of ensuing tumours are determined largely by the tissue of origin: mixed carcinomas with squamous and columnar components are found in the cervico-vaginal epithelium only, basal cell carcinomas arise mainly from the hair follicles of the vulva, sarcomas in the connective tissue. Squamous-celled tumours with varying degrees of keratinization occur both in the vulval region and the cervico-vaginal tract. Since no mixed carcinomas have been elicited in the cervix and no primary sarcomas in the vulva, the discussion deals mainly with the effect of castration and dosage variation on the induction of comparable squamous-celled tumours in the vulva and the cervico-vaginal tract and of sarcomas at the latter site. Basal-celled tumours of the vulva are not considered here.

The process of carcinogenesis of the epithelial tumours is essentially the same in the vulva and the cervico-vaginal region, passing from hyperplasia through radication, papillomas, microcarcinomas to the fully malignant state. The tumours are multifocal in origin and large " units " arise from the confluence of adjacent foci. Penetration of the panniculus carnosus has been abandoned as

FIG. 21.—Age-specific rates of induction by 40, 20, 10 or 5 weekly applications of DMBA of squamous-celled vulval tumours in intact rats.

*the* criterion of malignancy, since hair follicles in anagen may pass through it and since the cytological features of malignant tumours above and below this layer are identical. Similarly invasion is considered an important step in malignant progression, but is not as significant as the ability for continued proliferation of invading cells. Invading cells which keratinize in the stroma as they do in the microcarcinomatous stage, are not fully malignant and lack the quality of " xenoplasia ". This itself has various degrees in further progression as shown for instance by the discrepancy between the incidence of lymphatic and vascular permeation and embolism and that of metastatic deposits in regional nodes or at distant sites. This means that tumour cells capable of growing in the adjacent stroma and of invading vessels may not yet be able to grow in other environments, such as lymph nodes, lung, liver, etc.

Similar degrees in xenoplasia characterize the progression also of sarcomas which may extend widely by continuous growth, invade vessels and lymphatics and still produce metastatic deposits only relatively rarely. Whether sarcomas have a multifocal or at least multicellular origin like the epithelial tumours, cannot be proved. The early stages in their development are difficult to recognize and evaluate and the well-defined intermediate steps in the formation of carcinomas, *i.e.* radication, papillomas and microcarcinomas are missing. Continued

Fig. 22.—Age-specific rates of induction by 40, 20, 10 or 5 weekly applications of DMBA of squamous-celled vulval tumours in castrate rats.

proliferative activity with characteristic cytology and histology are the main criteria for the diagnosis of their malignancy with later on invasion of extra-territorial regions. The wide spectrum of histological features in a given sarcoma may be interpreted as evidence for multicellular origin with subsequent fusion or equally for the separation of different cell strains and clones from a single " multipotential " malignant cell. Neither castration nor variation in frequency of DMBA paintings appears to influence the histological type of sarcoma. The influence of varying the number of paintings on the induction of tumours in the cervico-vaginal tract and vulva can be seen in (a) the level of the threshold dose, (b) the increase in tumour incidence and shortening of the induction period with increasing number of doses, (c) the levelling off of tumour induction at certain dose levels, and (d) the appearance of an " optimal " dose, *i.e.* a decrease in tumour incidence from a peak level with increasing number of paintings.

The threshold dose for induction of sarcomas is relatively high (Fig. 11), low for epithelial tumours in cervix and vagina particularly of castrates (Fig. 14) and intermediate for those of intacts and in the vulva (Table II). Thus the sensitivity to the carcinogen is low for the cervico-vaginal stroma, intermediate for the vulval epidermis and high for the cervico-vaginal epithelium of castrates. The differences between intact and spayed rats suggest that hormonal influences affect the sensitivity which is thus not a fixed property of the tissues.

The increase in tumour incidence and shortening of the induction period with increased number of applications is shown in the incidence of sarcomas in intacts (Fig. 12 and 13) and up to 20 applications in castrates; it is also evident in the induction of vulval tumours (Fig. 19, 20, 21 and 22) and particularly for that of carcinomas (Table II). Though this correlation is by no means linear, increases in carcinogenic stimuli might be expected to increase the chances of malignant conversion in a given tissue by either acting as initiators plus promoters on single cells or by affecting a greater number of cells as must be the case in the multifocal origins of epithelial tumours. This correlation does not apply to all neoplasms and may hold only for certain ranges of dosage.

Thus for the induction of sarcomas in spayed rats there is a significant increase with dose up to 20 applications, but beyond this number there is, if anything, a fall in incidence (Fig. 11) and a slowing down in the rate of tumour induction (Fig. 12 and 13). Prolonging and increasing the number of applications during the lifetime of the rats, *i.e.* for up to 58 weeks, fails to raise significantly the incidence of tumours (Fig. 13). Sarcomas do not occur after an interval of 400 days (Fig. 12); this may imply that after a certain period resistance to carcinogenic stimulation supervenes under certain hormonal or environmental conditions, *i.e.* unless initiated cells have formed a tumour by a certain time they are later unable to do so. Such age-specific tumour incidences are known both for human pathology and for induced tumours (Doll, 1962; Lindop and Rotblat, 1962). An acquired tolerance for resistance to prolonged carcinogenic stimuli may account also for the levelling off in sarcoma induction in castrates.

Except for rats given 2 injections of oestradiol within 48 hours of birth and painted once weekly with acetone, no epithelial cervico-vaginal tumours have been observed in our strain of rats without the application of carcinogens (Cherry and Glucksmann, 1968). It can thus be assumed that at least one application of DMBA is required to induce such tumours though their percentage is not known. With 5 doses some 70% occur in castrates and about 20% in intacts. This level is maintained up to 20 doses but drops drastically with further applications particularly in spayed animals (Fig. 14–16). This fall cannot be accounted for by an increase in the incidence of sarcomas, since it increases in intact but not in castrate animals. Furthermore, the rate of induction of epithelial tumours and sarcomas up to 400 days is the same in castrates given 20 doses. There is thus no interference or competition for the development of these two tumour types. The incidence and rate of induction of vulval tumours shows no correlation with that of cervico-vaginal neoplasms and no interference with them. While the yield of papillomas and carcinomas in the cervix and vagina decreases with increased dosage, that in the vulva increases with greater number of applications.

The " optimal " dosage for the epithelial tumours of the cervix and vagina cannot be accounted for either by competition with carcinogenesis elsewhere, or by any shortening of the period of observation: the age-specific plots (Fig. 16) show that for the same time interval castrates given 20 doses have 4 times the percentage of tumours as those with 40 or more paintings. In intact rats fewer epithelial tumours are induced at low dose levels and the reduction in incidence by increased numbers of paintings is consequently not as drastic as in castrates.

Optimal dosages have been described for radiation induced tumours and one of the hypotheses put forward in explanation is that at high dose levels a " lethal " interferes with a " carcinogenic " action. This assumes that cells which might have given rise to cancers are killed by larger doses of radiation and thus the incidence of tumours decreases with increasing dose beyond a critical optimal value. There is no evidence for an increased lethal effect with dosage or rather number of applications of DMBA in the cervico-vaginal epithelium nor for any increase in " carcinogenic " potency in the range of 5 to 20 applications. If a lethal effect is assumed, it must affect potential stem cells for tumours selectively since there is no widespread ulceration or degeneration of the epithelium. In fact in castrate as well as in intact rats treated throughout life the cervico-vaginal epithelium is hypertrophic and often shows radication as evidence of proliferative activity. Furthermore, the presumed effect would apply to the cervico-vaginal epithelium and not to the vulval where the incidence of similar squamous-celled tumours increases with number of paintings.

The decreased incidence of epitheliomas with increased number of treatments might be explained by an inhibitory effect on the epithelium by the stroma changed by DMBA. Though this explanation cannot be excluded categorically, it is unlikely because in the castrate the stroma remains atrophic, while in the intact it is often increased and stimulated to sarcoma formation. Also in castrates treated intermittently with oestradiol or continuously with cholesterol and given the same regime of DMBA, the stroma remains atrophic though the incidence of epithelial tumours is greatly increased (Glucksmann and Cherry, 1968). It thus seems likely that the inhibitory effect of increased dosage is exerted directly on the cervico-vaginal epithelium, that it is concerned with the sensitivity to carcinogens and can be altered at least in castrates by additional treatments. The exact mechanism of this action remains to be elucidated.

Castration generally promotes the appearance of cervico-vaginal papillomas and carcinomas and inhibits that of sarcomas, though this statement has to be qualified to allow for variation with dosage of carcinogens. Thus at low levels of DMBA applications the threshold dose for sarcomas in castrates tends to be lower than in intacts and the rate of tumour induction is also faster. At the high dose level, however, the induction of sarcomas is depressed both in rate and percentage (Fig. 11 and 12). Again except for the highest dose level, the rate of induction and total incidence of epithelial tumours of the cervix and vagina is significantly increased in castrates (Fig. 14, 15 and 16). Though there is no apparent effect of castration on the total incidence of squamous-celled tumours of the vulva (Fig. 19–22), the progression to carcinomas occurs at lower dose levels in castrates (Table II). In discussing the effects of castration or alternatively that of additional oestrogenic medication on tumorigenesis it is essential to take into account the level of carcinogenic stimulation. While allowance must be made for species differences, reports of lower threshold levels for cervico-vaginal carcinomas in spayed mice (Murphy, 1961; Taki, 1967) and promotion of tumour formation by oestrogens in castrates treated for limited periods with carcinogens may be cited as evidence of the interdependence of carcinogenic dosage and hormonal status and stimulation of the target organs. It is also necessary to draw attention to the difference in structure of the target organ if painting rather than the impregnated thread method is used: in the former the vagina and exocervix are the principal targets, in the latter the endocervical region. While in the rat only intermittent oestrogenic stimulation promotes the induction of cervico-vaginal sarcomas and epitheliomas in castrates painted with DMBA throughout life, no such effect occurs in intacts. Whether a similar promotion can be obtained by continuous or intermittent oestrogenic stimulation at lower carcinogenic dose levels is being investigated now.

The authors are grateful to Dame Honor B. Fell, F.R.S., for reading the manuscript and to Mr. G. C. Lenney, A.I.S.T., for the illustrations.

### REFERENCES

ALAUDDIN, S. AND ZAMAN, H.—(1967) *Acta cytol.*, **11**, 211.
BARBIERI, G., OLIVI, M. AND PAOLETTI, I.—(1961) *Lav. Ist. Anat. Istol. patol Univ. Perugia*, **21**, 39.
BLANZAT, S., HIRAI, M. AND PINCUS, G.—(1966) *Abstr. Pap., 2nd Int. Congr. Hormonal Steroids*, Milan, p. 317.
CHERRY, C. P. AND GLUCKSMANN, A.—(1968) *Br. J. Cancer*, **22**, 728.
DOLL, R.—(1962) *Br. J. Radiol.*, **35**, 31.
GARDNER, W. U.—(1953) *Adv. Cancer Res.*, **1**, 173.
GLUCKSMANN, A. (1963) *Natn. Cancer Inst. Monogr.* No. 10, 509.
GLUCKSMANN, A. AND CHERRY, C. P.—(1958) *Br. J. Cancer*, **12**, 32.—(1962) *Br. J. Cancer*, **16**, 634—(1968) *Br. J. Cancer*, **22**, 545.
ISLAM, K. M. N. AND ZAMAN, H.—(1965) *Acta cytol.*, **9**, 446.
KASLARIS, E. AND JULL, J. W.—(1962) *Br. J. Cancer*, **16**, 479.
KLAVINS, J. V. AND KAUFMAN, N.—(1962) *Acta cytol.*, **6**, 267.
KRIEG, A. F. AND REAGAN, J. W.—(1961) *Lab. Invest.*, **10**, 581.
LAFFARGUE, P., SAMSO, A., LUSCAN, R. AND FRANCOIS, H.—(1965) *Annls Anat. path.*, **8**, 85.
LINDOP, P. J. AND ROTBLAT, J.—(1962) *Br. J. Radiol.*, **35**, 23.
MEISELS, A.—(1964) *Acta cytol.*, **8**, 274.—(1966) *Cancer Res.*, **26**, 757.
MUEENUDDIN, G. AND ZAMAN, H.—(1967) *Acta cytol.*, **11**, 205.
MURPHY, E. D.—(1961) *J. natn. Cancer Inst.*, **27**, 611.
TAKI, I.—(1967) 'Uterine Carcinogenesis and Hormonal Imbalance'. *Jap. Obstet. Gynaec. Soc. Monogr.*
THIERY, M.—(1963) 'Het Experimentele Carcinoma Colli Uteri'. Brussels (Arscia & Presses Academiques Europeenes).
THIERY, M. AND VAN GIJSEGEM, M.—(1965) *Br. J. Cancer*, **19**, 418.
VELLIOS, F. AND GRIFFIN, J.—(1957) *Cancer Res.*, **17**, 364.